# Maintenance and Quality Control of Medical Equipment Based on Information Fusion Technology

**DOI:** 10.1155/2022/9333328

**Published:** 2022-10-13

**Authors:** Jiansheng Li, Yajie Mao, Jin Zhang

**Affiliations:** ^1^Shanxi Bethune Hospital, Shanxi Academy of Medical Sciences, Tongji Shanxi Hospital, Third Hospital of Shanxi Medical University, Taiyuan 030032, China; ^2^Tongji Hospital, Tongji Medical College, Huazhong University of Science and Technology, Wuhan 430030, China

## Abstract

In the medical field, to ensure the use of large medical equipment, it is necessary to carry out regular maintenance on large medical equipment. In the process of maintenance and maintenance of large-scale medical equipment, most medical personnel have not established a corresponding quality management system, neglecting daily maintenance and maintenance, resulting in many hidden dangers of medical accidents. To this end, the quality control of large medical devices should be strengthened, the control before, during, and after the event should be done well, and a comprehensive analysis of the operation methods of the equipment should be carried out to achieve reasonable maintenance of the equipment. Therefore, this paper discusses the maintenance, management, maintenance, and quality management of large medical equipment under the function of information fusion technology. This paper summarizes the problems encountered in the maintenance of medical equipment in the past and creates a medical quality control system to manage the maintenance and quality control of medical equipment. In the maintenance system of medical equipment, scientific management theories and methods are used to predict, adjust, inspect, and account for the quality of the entire production process of the equipment, and establish a complete quality monitoring and management system. To achieve optimal maintenance and economic benefits, the overall quality of medical equipment can be comprehensively improved. The data shows that the actual number of monitors for quality control testing in 2020 is 502 units, 496 units have passed the initial inspection, and 502 units have passed the maintenance, which shows that the maintenance and quality control of medical equipment based on information fusion technology is effective.

## 1. Introduction

The survival and development of a hospital is inseparable from medical facilities. Strengthening the repair and maintenance of medical equipment can ensure the normal operation of the hospital and reduce medical expenses. With the use of medical devices, the hospital's treatment methods have also been further improved. In practical applications, due to the automation and complexity of medical devices, maintenance must change past habits during use. Maintenance personnel must regularly check related equipment, which puts forward higher requirements for the maintenance and management of medical equipment. Therefore, this paper discusses the maintenance and repair of large medical equipment. The maintenance and management of medical equipment ensures its superiority in use. The clinical application of medical equipment is essential, and its maintenance quality directly affects the work efficiency of the hospital, as well as the survival and health of patients. Therefore, it is necessary to carry out routine maintenance on existing medical devices and analyze their scientific and institutionalized advantages in operation, thereby greatly improving the service life of medical devices.

Research into the maintenance and quality control of medical equipment has been ongoing. Zheng discussed how to optimize the quality control of medical devices and improve the implementation effect of quality control, to improve the quality of medical devices and the level of clinical diagnosis and treatment [1]. Hao discussed the use of the Medatc system for inspection and failure statistics of hysteroscopic and laparoscopic equipment [2]. Saffarpur aimed to evaluate the effectiveness of curing light equipment routinely used in Karaj Dental Clinic in 2016 [3]. Jun evaluated the current situation and the current situation of quality control in the intensive care unit (ICU) of Sichuan Provincial Hospital of Integrated Traditional Chinese and Western Medicine and Minority Hospital [4]. Jose developed a series of technical quality control (TQC) guidelines for radiotherapy equipment [5]. Liu Y reviewed the current status and future trends of clinical quality control procedures and MRI equipment regulation [6]. The data information obtained by these studies is too simple, and the data is not integrated, so the value of the data cannot be fully utilized.

Many scholars have conducted research on information fusion technology. Du used the big data association rule mining method to realize the information fusion of mathematics teaching resources [7]. Zhang explored the potential of multisource information fusion techniques to improve model calibration and prediction performance in Chinese herbal medicine extraction process [8]. Shuai designed a fusion method of radar and electro-optical information [9]. These research fields do not cover the medical field, and there is also a lack of data support, so this paper studies the maintenance and quality control of medical equipment based on information fusion technology.

This paper takes several common medical equipments in a hospital as the research object and uses the equipment qualification rate as the research index to test the qualification rate before and after equipment maintenance. The data shows that the actual number of defibrillators tested for quality control in 2018 was 19, of which 16 passed the initial inspection and 18 passed the maintenance, indicating that equipment maintenance and quality control play a significant role in medical equipment.

## 2. Medical Equipment Maintenance and Quality Control

**Figure 1:**
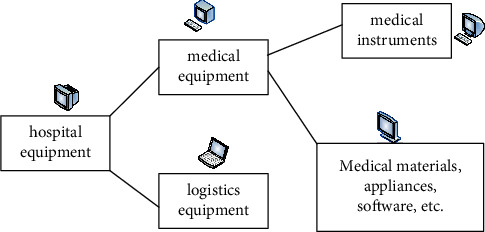
Conceptual figures related to medical devices.

Medical equipment is different from both hospital equipment and medical equipment. Hospital equipment mainly includes two major parts: medical equipment and logistics equipment. Medical equipment and medical equipment include medical equipment and medical materials, appliances, software, etc. The conceptual relationship related to medical equipment is shown in Figure 1.

Large-scale hospital equipment maintenance needs to be improved:

The poor management of the acceptance of large medical devices has seriously affected the follow-up operation of the hospital. For example, in the purchase of large medical equipment, the hospital management model has some flaws and defects [10, 11].

In the daily procurement, the large-scale equipment of the hospital is generally carried out by contract and then allocated according to the corresponding procedures. Due to the inability to carry out centralized management, it will cause some negligence of the distribution personnel during the transportation process, resulting in insufficient overall maintenance capability of the equipment [12, 13].

During the acceptance process, because the hospital did not conduct inspections in time, the potential risks of medical devices could not be found in time. This has a negative impact on the subsequent use and maintenance of medical devices, thereby affecting the normal use of medical devices [14, 15]. In addition, in the management of equipment, it has not reached the corresponding standard, and there is a big deviation. In addition, due to the unsound management mechanism of large medical equipment in the hospital itself, medical staff cannot perform effective operations when using them [16, 17].

Many medical workers do not have a good attitude towards instruments in their daily inspection and use [18, 19]. They thought that once a medical device was damaged, it would be repaired by the financial department of the hospital, not regularly repaired, nor regularly inspected according to the regulations. This leads to overloading of many devices, which shortens the life of the device. Once a fault occurs, it is difficult to find relevant information, and it is difficult to find the personnel responsible for it, which affects the information management level of the hospital and thus affects the subsequent development of the hospital. Therefore, in the future work, to realize the comprehensive development of the hospital, the first problem to be solved is the information management [20, 21].

The elements of medical equipment maintenance are as follows:

The essence of the maintenance of medical equipment is to prolong the service life of the instrument, ensure the integrity and utilization rate of the instrument, and reduce the probability of failure during use [22]. In the maintenance department of the hospital, according to the characteristics and structure of the equipment, a new equipment maintenance plan was designed to ensure the safety and operation of the equipment. In the traditional security measures, the following methods can be used to optimize, such as regular inspections. In the daily inspection, regular maintenance of the instrument is also an important basis for equipment maintenance. Therefore, it is the key to ensure the safe operation of equipment to effectively prevent equipment safety accidents and to carry out regular maintenance and repairs.

Regular maintenance and preventive maintenance of the machinery are carried out on a regular basis to ensure that the machine can be cleaned regularly according to the technical requirements of the machine during normal operation. Then, perform a performance test. Detect damaged parts of machinery and equipment, analyze the overall condition of lines and waterways, and centrally optimize the operation of large medical equipment to ensure that both are fully functional.

To ensure the efficient development of the hospital, the equipment of the general department must be standardized and perfected and people-centered. Pay full attention to the improvement of the quality and skills of maintenance personnel, stimulate their enthusiasm for work, let them be assertive, take the patient as the center, take the survival and development of the hospital as their own responsibility, and adhere to the interests of society. Optimize the financial responsibility of the hospital to ensure that it can obtain new competitive forces in the market competition and achieve all-round development.

The quality control of large medical devices is as follows:

1. Regularly organize training
2. For the current medical equipment management, we ensure that the employees can consciously carry out the maintenance of medical equipment. For example, managers have to change their minds and adapt to large-scale medical devices. According to their professional skills and business level, choose excellent managers. In addition, after new equipment is put into service, employees must be regularly evaluated and trained. Equipment such as B-ultrasound and CT need to be licensed. After that, a responsibility control system was implemented for the quality inspection of the equipment, so that the defect behavior of the equipment was effectively implemented. At the same time, punish the causes of medical device failure due to personal reasons, urge operators to operate in accordance with standards, and improve the use, quality, and management level of medical devices.
3. Implement the quality management system of equipment
4. Implement the equipment quality control system in the standardized management, strengthen the large-scale medical equipment on the basis, improve the process of pre-purchase, in-purchase, use, etc., establish a set of evaluation systems and systems, and supervise the quality of medical equipment. For example, consider the entire quality management as the most critical step in the purchasing process. Pay more attention to relevant personnel and improve the quality of the overall equipment. To ensure the quality of medical devices, hospitals must conduct quality control and monitoring on a regular basis. Carry out a comprehensive inspection of the maintenance, installation, and debugging of the equipment, debug when purchasing the equipment, check the consistency of the equipment, and analyze the influencing factors in use to improve the quality of the equipment. For example, during use, the operator's working methods can be standardized, regular training can be organized, and comprehensive use and records of equipment, including maintenance records and maintenance records, can be done well. Ensure the timely control of existing problems and do a good job in the information management of large medical devices. With the continuous improvement of the level of medical informatization, the hospital must carry out a comprehensive management model reform, obtain new failure rates and maintenance rates, and conduct data analysis on them. In terms of medical device management, the latest mobile software technology and RFID technology are adopted to realize the monitoring and management of medical devices and ensure that the quality of large medical devices has reliable support.

**Figure 2:**
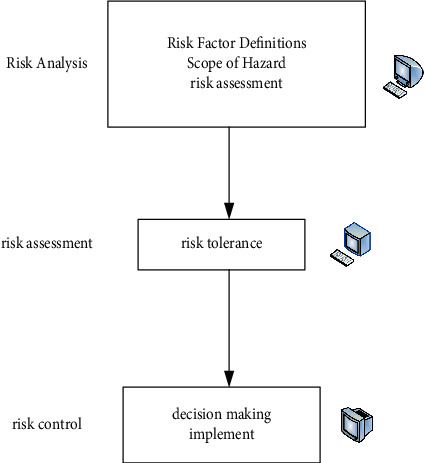
Risk assessment model.

Determine the quality assurance measures that should be taken according to the risk level of the equipment. During the implementation process, the effectiveness of risk control should be dynamically evaluated, and the risk estimation and risk analysis should be adjusted repeatedly. The risk assessment model is shown in Figure 2.

Risks are divided into six parts: equipment attributes, physical risk, equipment characteristics, safety performance, lethal state, and frequency of use.

The equipment properties and their risk scores are shown in Table 1.

Physical risk mainly refers to the adverse consequences that may be caused by abnormal operation of the equipment, which can be summarized as death, injury, errors, etc. The physical risks and their risk scores are shown in Table 2.

The equipment characteristics and their risk scores are shown in Table 3.

The safety performance and its risk score are shown in Table 4.

The lethal state and frequency of use are shown in Table 5.

**Figure 3:**
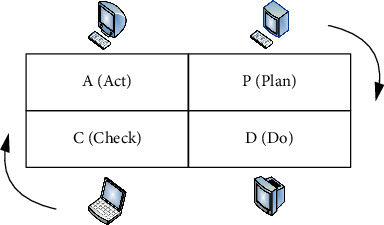
PDCA cycle.

The meaning of PDCA cycle is to divide quality management into four stages, namely, Plan (planning), Do (execution), Check (check), and Act (processing). In the quality management activities, it is required to make plans, implement the plans, and check the effect of implementation. Then, the successful ones are included in the standard, and the unsuccessful ones are left to the next cycle to solve. The quality control method adopts the PDCA cycle management method, as shown in Figure 3.

The overall level and ability of medical equipment control is concentrated in the medical equipment quality control system, which plays a great role in the management and control of hospital medical equipment, and depends on it to improve the overall medical level of the hospital. Hospitals not only need to closely integrate the construction of the medical equipment quality control system with their own characteristics, and learn from the research of scholars, but also follow the following principles:

Practical principles. All indicators of the medical equipment quality control system need to collect data in the form of questionnaires and then process the questionnaire data. Therefore, the index system must be practical, can truly reflect the overall level of medical equipment, and be easy to operate.

Scientific principles. The overall framework of the paper, the research objects, and the guiding methods are the cornerstones, and the index construction of the medical equipment quality control system can be scientific, rational, and justified.

Quantitative principles. The construction of the medical equipment quality control system is to prepare for later empirical research, so each index in the system needs to be mathematically quantified, to carry out the modeling and quantitative analysis of the empirical research.

In general, the life cycle of the designed equipment is determined by the quality of the medical equipment. Therefore, this paper first chooses the life cycle theory and the total quality management theory to select the indicators. Then, combine the previous research to classify the indicators, and finally determine the overall system indicators of medical equipment quality control combined with the risk management theory.

The existing problems in the quality control of hospital medical equipment are:

Insufficient quality inspection personnel. A common phenomenon in hospitals now is the lack of professionals. Many medical engineering technicians and some other medical personnel take into account the measurement and quality control work, which originally required professionals to be responsible. Because of the limited number of engineering and technical personnel and the large number of medical facilities, these technicians are usually only responsible for the management and maintenance of the facilities. This has led to problems such as overdue use and missed inspections in many facilities. Not only that, but some facilities have lost quality control.

Acceptance testing is not standardized enough. Hospitals only rely on personal feelings and experience to test whether newly purchased medical facilities are qualified. When the facility is checked and accepted, it is still the traditional way to check whether the outer packaging of the facility is in good condition, whether the relevant accessories are sufficient, and whether it can be powered on normally. This kind of verification to judge whether a facility is qualified or not based on simple external performance is seriously insufficient. There is no scientific and accurate reference index and verification basis for this method. In this way, the purchased equipment is often unqualified, and it is easy to make mistakes and dangers in clinical use.

The purpose of the construction of the medical equipment quality control system is to ensure that the stability, safety, and accuracy of the parameters of the equipment can be guaranteed. The specific work mainly includes the following three parts: The first is to ensure the formation of the organizational management system. Form a situation in which special personnel are responsible and leaders pay attention. And increase the supervision and management of the entire process, methods, and other links. Second, building a quality control process system can ensure that quality management is implemented in the entire life cycle of the equipment, and it can be continuously improved and improved in actual use. The third is the construction of the security system. Strengthen the allocation of special tools and professional testing personnel, to form a guarantee for the good operation of the quality control system.

**Figure 4:**
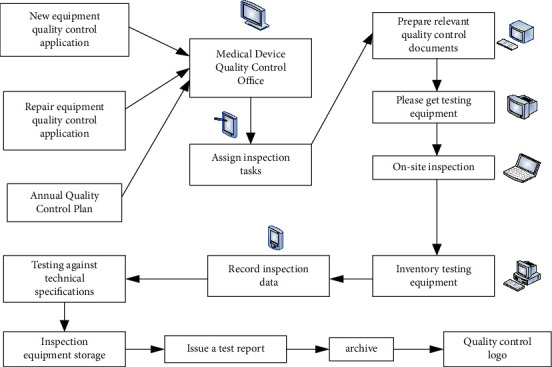
Medical device quality control system.

The medical equipment quality control system is shown in Figure 4.

The so-called quality inspection and control before the application of equipment is not only necessary to determine the comprehensiveness of its safety performance before the purchase of medical equipment, but also to sign an effective safety contract to ensure the quality level of the entire purchased medical equipment. At the same time, this is also the original step of equipment quality assurance.

Project demonstration quality control. In each department of the hospital, as long as there is a need for some high-risk medical equipment or equipment, professional project approval for the required equipment, as well as a wealth of data demonstration, must be carried out. At the same time, according to the relevant research data, we set up a special project report and establish a file. The main items that should be included are: all information and details related to the required medical equipment, and all feasibility assumptions related to the required equipment, as well as feasibility studies. At the same time, according to the risk and value of the required medical equipment, the setting and the reason for the need should be justified.

Plan approval for quality control. In the gradual development stage of science and technology, the detection and control of the safety factors of medical equipment and equipment in major hospitals is the primary prerequisite for medical development. At the same time, it is also an important basic prerequisite for adapting to the development of science and technology. Therefore, for the safety guarantee of medical equipment, it is necessary to carry out the planned quality inspection and control for approval. In terms of medical equipment, risks in the process of its use should be avoided, and procedures and projects that require technical content should be encouraged and supported, and attention should be given to action. For some more important and high-risk medical devices, it is necessary to conduct in-depth investigation and research by multi-level leaders and departments, as well as relevant high-level technical personnel. After obtaining the plan approval, it can be effectively implemented and used, to effectively control the quality and safety of plan approval.

Manufacturer selection quality control. For the purchase and investment of medical equipment required by the hospital, it is necessary to go through very strict procedures and steps to check and test the quality of the required products and equipment. Among them, first of all, it is necessary to confirm the relevant certificates of the purchased medical equipment manufacturers, for example, similar certificates such as the equipment registry and the manufacturer's authorization letter, must be strictly checked and tested. Secondly, it is to carry out careful measurement and evaluation of relevant health aspects. At the same time, health testing is also the most basic control and testing for the medical quality of medical manufacturers. Then, for the selection of medical equipment manufacturers and suppliers, the selection of medical equipment manufacturers should be determined according to the basic conditions of the manufacturers, such as safety and quality issues, and hygiene and technical aspects. Secondly, according to the various departments in the hospital, the equipment required, and the data displayed in the database, all manufacturers should be screened, to effectively complete the quality screening of medical equipment.

Business negotiation quality control. Regarding the selection of the medical equipment required by the hospital, at the stage of negotiation, the hospital's bidding form and the determination of the selection method should be used to negotiate the selection of medical equipment to achieve its quality control. To better ensure the safety, quality, and level of medical equipment, it is necessary to carry out strict and formal negotiation procedures and steps, that is, relevant personnel from many departments of the hospital should be integrated into the negotiation process.

Contract quality control. At present, the operation and development of many enterprises are inseparable from the establishment of contracts, and the same is true for hospitals. Therefore, in the purchase and setting of the required medical equipment, an effective, fair, and just contract should be established in accordance with the corresponding laws and regulations to carry out quality supervision and risk control. During the establishment of the contract, not only the approval of the relevant departments, but also more professional lawyers and personnel must be recruited to set the procurement standards. As well as the writing of the contract, from the establishment of the contract, as well as the approval and testing of the relevant hospital leaders and departments, the quality assurance and risk detection and control of the purchased necessary medical equipment.

Installation acceptance quality control. After purchasing, reviewing, negotiating, and signing the contract for the relevant medical equipment required by the hospital, the preordering activities of the medical equipment required by the hospital department and department are completed. The next step is the installation and acceptance of the overall equipment and the setting of the process. In the process of this step, the most critical and main problem is that the entire installation, configuration, and inspection need to be evaluated and tested to ensure the most basic safety and hygiene.

During the whole acceptance process, the supplier of medical devices should negotiate with the relevant departments and management personnel in the hospital to verify. For the purchased medical equipment, according to the testing of relevant national standards and the evaluation, standards of the military, quality control, and evaluation are carried out to fundamentally complete the evaluation of the quality and level of the equipment required by the hospital in terms of installation and acceptance. On the other hand, in this step, the risk detection of medical equipment, as well as the control of quality and level. It is also for the hospital to be able to cause safety problems and quality risks in the process of using the equipment in the future, resulting in some important medical failures and accidents.

The hospital selects and purchases the required medical equipment in a timely manner so that it can be used in the hospital. Then, in the whole process of using medical equipment, medical staff and various departments should use and operate them in strict accordance with the settings and standards of medical equipment. However, in the process of use, relevant personnel and department personnel should also carry out irregular and irregular quality inspection and inspection according to the purpose of the device. In this way, it can not only carry out quality control and risk prevention, but also find and solve problems in time.

In the hospital, regarding the use of medical equipment, it is necessary to carry out the clinical implementation and application of its equipment according to relevant regulations and reasonable suggestions, and to ensure safety issues and control the quality and level. With the development of the medical industry, major hospitals and related health departments, as well as managers of medical associations, have increased their awareness and attention to the safety of medical devices. It has also formulated relevant regulations and systems for the clinical implementation of medical equipment and equipment, the control of risk factors, and the prevention of quality. Faced with various problems and deficiencies in the process of clinical trials, diversified management methods and systems should be used to prevent and control risks.

In hospitals, many medical staff need to strengthen standardized actions and operating procedures for the use of related clinical equipment and product operations to improve the hospital's clinical implementation technology and efficiency for new medical equipment. Based on this, the hospital should provide the most basic and standardized training for the relevant medical staff in the hospital and the users of equipment at all levels. It is necessary to train not only the methods and procedures of the newly added equipment, but also the nursing methods of the relevant personnel, so as to fundamentally guarantee and control their quality.

One is equipment installation and acceptance training. The relevant new equipment users should be in the installation and acceptance stage of the new equipment, as well as the important control and debugging stage. To understand and learn the operation method of the equipment is not only very helpful for subsequent clinical experiments; at the same time, it can also learn a lot of equipment maintenance and repair knowledge, which is convenient for the protection of new equipment and reduces the safety risk of the equipment. The second is annual equipment use training. The hospital should conduct annual training on the operation and use of equipment for all doctors and related medical staff who can access and use the new equipment. At the same time, the hospital should also integrate the newly added clinical equipment, its use and operation, and the functions and skills of the relevant equipment into the assessment content according to the annual assessment of all medical staff. This can not only strengthen the medical staff's understanding and awareness of high-risk equipment, but also promote the hospital's quality control and management of these equipments. The third is to formulate operating procedures. Hospitals have complete procedures and procedures for the use and operation of medical equipment, which must be effectively implemented and managed. In addition to posting on the wall the use and control procedures of medical devices, the hospital also requires medical staff to carry out the steps of cleaning and testing high-risk devices one at a time, thereby reducing the quality risk of medical devices.

According to the legal systems and rules and regulations related to the hygienic use of medical devices and clinical operation issues proposed by the relevant medical departments and health management and assessment departments, that is, the strategy of the “Measures for the Management of Instruments and Equipment in Medical and Health Institutions” to fundamentally restrict and manage the relevant hospital medical staff and the implementation and operation of clinical equipment to improve the quality supervision and risk prevention and management of the overall equipment.

One is to insist on working with licenses. Hospitals should carry out strict handling and assessment of hospital medical staff who contact and use high-risk medical devices. After completing the prescribed standard tasks and passing, the clinical implementation and operation of medical devices can be carried out. Even for larger volumes such as ultrasonic medical equipment and DSA, it is necessary to pass the training and assessment of relevant physician certificates, and only after obtaining a formal employment certificate can the equipment be applied and controlled to ensure the quality management and risk supervision of medical equipment. The second is to establish a system of persons responsible for the use of medical equipment. The management personnel of the relevant departments of the hospital, as well as the medical equipment supervisors, shall register the persons responsible for the use and manipulation of the hospital medical equipment, form files and books, and register and manage their implementation and manipulation to ensure the quality control of the equipment.

In the clinical trial of the hospital, the regular maintenance and testing of medical equipment is a key step in its clinical implementation and application, and it is also the most important factor affecting the risk of medical equipment use. First, we do preventive maintenance for all staff. For the control and application of hospital medical equipment, not only the maintenance and management of managers is required, but also various departments and departments are required to carry out effective prevention and maintenance. It can be divided into departments or levels, and all staff participate in the maintenance, consideration, and testing of medical and health equipment, fundamentally improve the quality management and control of medical equipment, and avoid the emergence of high-risk risks. Second, emergency maintenance quality control. As a medical device commonly used in hospitals, risks and failures will occur from time to time. Therefore, the hospital should, according to this situation, formulate an all-day duty model and plan for the relevant maintenance department of the hospital, and let the maintenance workers work 24-hour shift mode to wait for the maintenance of the hospital's medical equipment at any time. When receiving a request for declaration of equipment maintenance, the medical equipment must be repaired in the shortest possible time to avoid more serious equipment failures. Fundamentally, the quality of medical equipment can be effectively controlled to prevent the occurrence of more serious risks. Finally, quality control after maintenance. To fully ensure the safety of equipment quality, a series of strict inspections and correlation tests must be carried out after handling equipment failures. All personnel related to the equipment should attach great importance to safety issues. After repairing the equipment that has been in the category of mandatory measurement and testing, and strictly inspecting the quality of all aspects of the medical equipment in time, only the equipment that has passed the overall quality can be officially used.

Scrap standard quality control. Under the specific service period stipulated by the relevant system, if the medical equipment still cannot be used normally or is lower than the normal quality standard after being repaired by the professional department, or equipment whose repair cost is too expensive or even exceeds the specified repair cost the standard can be applied for normal scrapping. However, the problems caused by real equipment are often intertwined and complex, and specific details such as the probability of equipment use, the objective environment of use, and maintenance will also have a great impact on the service life of the equipment. Instruments and equipment that have reached their end of life are widely available in practical work. However, because the relevant staff can properly operate and maintain the equipment, even if the equipment has exceeded the specified period of use, it still has a high operating quality. However, at the same time, many departments actively concealed the scrapping of equipment under the constraints of different factors.

Quality control of end-of-life equipment disposal. Relevant approval procedures are the directions that must be adhered to in the process of disposal of scrapped equipment. At the same time, the lasting use of relevant parts should be maintained as much as possible under the guidance of the idea of thrift first. The funds obtained after the scrapping process should be recorded in detail and reported to the financial department in the name of the funds for repairing and purchasing the provident fund.

This paper is based on the information fusion technology to realize the research on the maintenance and quality control of medical equipment. Measuring the information data *δε*(ϱ)(ϱ=1,2, ..., *γ*) obtained at time ϱ and positioning it on the number axis, the absolute distance between the information data *δε*(ϱ) and *δζ*(ϱ) is set as:

(1) $$\begin{array}{c} \eta \left(\varrho \right)=\left|\delta \varepsilon \left(\varrho \right)-\delta \zeta \left(\varrho \right)\right|. \end{array}$$

The distance between *δε*(ϱ) and all information data is

(2) $$\begin{array}{c} \theta \left(\varrho \right)=\overset{\gamma }{\underset{\zeta =1}{\sum }}\eta \varepsilon \zeta \left(\varrho \right). \end{array}$$

The average distance between all informative data is

(3) $$\begin{array}{c} \bar{\epsilon \left(\varrho \right)}=\overset{\gamma }{\underset{\varepsilon =1}{\sum }}\theta \left(\varrho \right). \end{array}$$

The set of all valid information data falling in the neighborhood is set as *ι*, if *ϵ*(ϱ) satisfies the following conditions:

(4) $$\begin{array}{c} \theta \left(\varrho \right)\geq \bar{\epsilon \left(\varrho \right)}\left(\forall \delta \varepsilon \left(\varrho \right)\notin \iota \right). \end{array}$$

Then, this set is called the optimized fuzzy set.

The fusion degree of *δε*(ϱ) and *δζ*(ϱ) is

(5) $$\begin{array}{c} \kappa \varepsilon \zeta \left(\varrho \right)=\exp\;\left\{-\frac{1}{2}\left|\delta \varepsilon \left(\varrho \right)-\delta \zeta \left(\varrho \right)\right|\right\}. \end{array}$$

Data fusion degree matrix:

(6) $$\begin{array}{c} \kappa =\left[\begin{array}{cc} \begin{array}{c} 1\\ \kappa_{21}\left(\varrho \right) \end{array} & \begin{array}{c} \begin{array}{ccc} \kappa_{12}\left(\varrho \right) & \ldots & \kappa_{1\alpha }\left(\varrho \right) \end{array}\\ \begin{array}{ccc} 1 & \ldots & \kappa_{2\alpha }\left(\varrho \right) \end{array} \end{array}\\ \begin{array}{c} \vdots \\ \kappa_{\alpha 1}\left(\varrho \right) \end{array} & \begin{array}{c} \begin{array}{ccc} \vdots & \ddots & \vdots \end{array}\\ \begin{array}{ccc} \kappa_{\alpha 2}\left(\varrho \right) & \ldots & 1 \end{array} \end{array} \end{array}\right]. \end{array}$$

The consistency fusion degree of sensor *δε* at time ϱ is

(7) $$\begin{array}{c} \lambda \varepsilon \left(\varrho \right)=\overset{\alpha }{\underset{\zeta =1}{\sum }}\kappa \varepsilon \zeta \left(\varrho \right)/\alpha . \end{array}$$

The distribution equilibrium is

(8) $$\begin{array}{c} \mu \varepsilon \left(\varrho \right)=1/\overset{\alpha }{\underset{\zeta =1}{\sum }}\left(\lambda \varepsilon \left(\varrho \right)-{\kappa \varepsilon \zeta \left(\varrho \right)}^{2}\right)/\alpha . \end{array}$$

The weight factor is

(9) $$\begin{array}{c} \nu \varepsilon \left(\varrho \right)=\lambda \varepsilon \left(\varrho \right)*\mu \varepsilon \left(\varrho \right). \end{array}$$

Normalized to get:

(10) $$\begin{array}{c} \xi \varepsilon \left(\varrho \right)=\nu \varepsilon \left(\varrho \right)/\overset{\alpha }{\underset{\varrho =1}{\sum }}\nu \varepsilon \left(\varrho \right). \end{array}$$

The fusion result is

(11) $$\begin{array}{c} \hat{\rho }=\overset{\alpha }{\underset{\varrho =1}{\sum }}\xi \varepsilon \left(\varrho \right)\delta \varepsilon \left(\varrho \right)=\overset{\alpha }{\underset{\varrho =1}{\sum }}\frac{\nu \varepsilon \left(\varrho \right)\delta \varepsilon \left(\varrho \right)}{\overset{\alpha }{\underset{\varrho =1}{\sum }}\nu \varepsilon \left(\varrho \right)}. \end{array}$$

Weighted evidence indicates the predictive power of the independent variable relative to the dependent variable. Weighted evidence:

(12) σςτς=σϱτϱ1≤ϱ≤αmax.

Taking the weighted evidence as a reference and the global credibility of the evidence as the basis for measuring the weight coefficient of this piece of evidence, the weight coefficients of other evidence can be expressed as:

(13) φϱ=σϱτϱσϱτϱ1≤ϱ≤αmax.

The basic confidence function is assigned as:

(14) $$\begin{array}{c} \chi \varepsilon \left(\varrho \right)=\beta \varepsilon \left(\varrho \right)/\overset{\alpha }{\underset{\varrho =1}{\sum }}\beta \varepsilon \left(\varrho \right). \end{array}$$

## 3. Maintenance Test

By establishing a quality control system for medical equipment, the stability and reliability of medical equipment have been improved year by year. This paper makes a statistical analysis of the data of hospital measurement and quality control in a hospital, and the analysis results are as follows.

**Figure 5:**
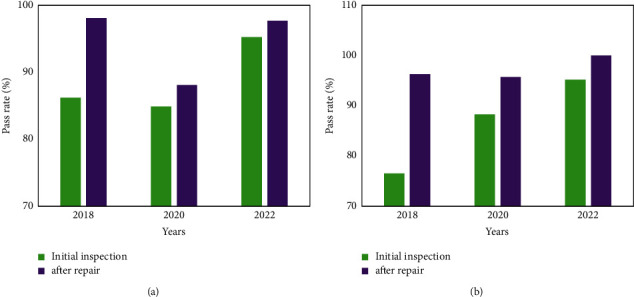
Quality control test results of sphygmomanometer and scale. (a) shows that the initial inspection pass rate of the sphygmomanometer in 2018 was 86.21%, and the pass rate after maintenance was 98.05%. The pass rate of initial inspection in 2020 is 84.86%, the pass rate after maintenance is 88.10%, the pass rate of preliminary inspection in 2022 is 95.25%, and the pass rate after repair is 97.68%. (b) shows that the first pass rate of the quality control test of the scale in 2018 was 76.52%, and the pass rate after maintenance was 96.26%. The pass rate of initial inspection in 2020 is 88.25%, the pass rate after maintenance is 95.70%, and the pass rate of preliminary inspection in 2022 is 95.21%, and the pass rate after repair is 100%. The qualified rate of the sphygmomanometer and the weight scale has been significantly improved after maintenance. It can be seen that the hospital equipment has been effectively improved after the practice of the medical equipment quality control system.

The quality control test results of the sphygmomanometer and the weight scale in 2018, 2020, and 2022 are shown in Figure 5(a) and Figure 5(b), respectively.

**Figure 6:**
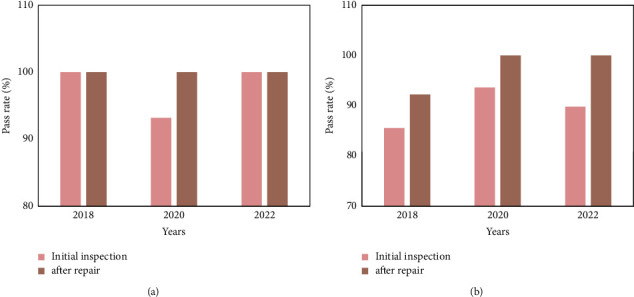
Quality control test results of electrocardiograph and B-ultrasound. (a) shows that the initial inspection pass rate of the ECG machine in 2018 was 100%, and the pass rate after maintenance was 100%. The pass rate of the initial inspection in 2020 is 93.18%, the pass rate after maintenance is 100%, and the pass rate of the initial inspection in 2022 is 100%, and the pass rate after maintenance is 100%. (b) shows that the initial inspection pass rate of B-ultrasound quality control inspection in 2018 was 85.56%, and the pass rate after maintenance was 92.22%. The pass rate of initial inspection in 2020 is 93.63%, the pass rate after maintenance is 100%, and the pass rate of preliminary inspection in 2022 is 89.80%, and the pass rate after repair is 100%.

The quality control test results of the electrocardiograph and B-ultrasound in 2018, 2020, and 2022 are shown in Figures 6(a) and 6(b), respectively.

**Figure 7:**
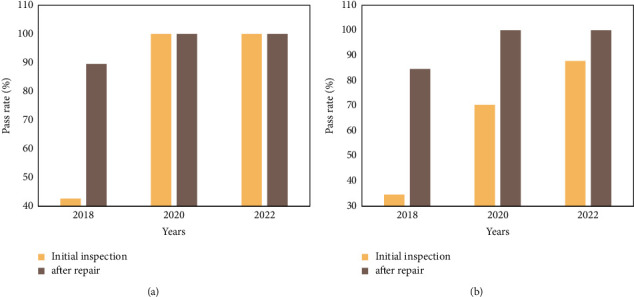
Quality control test results of ventilator and anesthesia machine. (a) shows that the initial inspection pass rate of the ventilator in 2018 was 42.64%, and the pass rate after maintenance was 89.52%. The pass rate of the initial inspection in 2020 is 100%, the pass rate after maintenance is 100%, and the pass rate of the initial inspection in 2022 is 100%, and the pass rate after maintenance is 100%. (b) shows that the initial inspection pass rate of the quality control inspection of the anesthesia machine in 2018 was 34.60%, and the pass rate after maintenance was 84.60%. The pass rate of initial inspection in 2020 is 70.32%, the pass rate after maintenance is 100%, and the pass rate of preliminary inspection in 2022 is 87.78%, and the pass rate after repair is 100%.

The quality control test results of the ventilator and anesthesia machine in 2018, 2020, and 2022 are shown in Figures 7(a) and 7(b), respectively.

**Figure 8:**
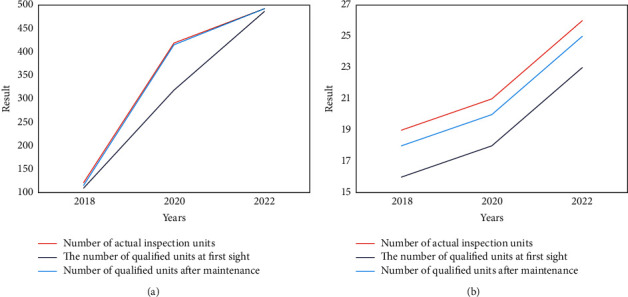
Quality control test results for monitors and defibrillators. (a) shows that the actual number of monitors in the quality control test in 2018 was 122, of which 100 passed the initial inspection and 116 passed the maintenance. (b) The actual number of defibrillators in the quality control inspection in 2022 is 26, of which 23 have passed the initial inspection and 25 have passed the maintenance. To sum up, no matter what kind of medical equipment, it is fully maintained after repair and quality control, thus prolonging the service life of the equipment.

The quality control test results of the monitor and defibrillator in 2018, 2020, and 2022 are shown in Figures 8(a) and 8(b), respectively.

## 4. Conclusion

The quality management of medical devices is an important part of hospital management, and it is also an inevitable trend of hospital scientific and technological progress. By establishing the quality management system of medical equipment, combining it with the thought of management, and applying it to clinical management. Quantitatively and qualitatively analyze the reliability, maintainability, and security of the instrument, so that the quality of the medical device can meet the predetermined or potential demand. And combine quantitative and qualitative requirements into technical indicators, to give full play to the value of medical equipment. In addition, this paper analyzes the problems existing in the maintenance and quality control of existing medical equipment and gives three suggestions before, during, and after the maintenance to optimize the maintenance process. At the same time, the PDCA cycle method is used to control the quality of medical equipment. The medical equipment maintenance and quality control system is immature and cannot be applied to major medical systems. Therefore, it is necessary to improve the system to the extent that it can be widely used.

## Figures and Tables

**Table 1:** Device properties.

Attributes	Score
Life support	12
Therapeutic equipment	6
Monitoring equipment	5
Equipment for diagnosis	3
Attributes	Score
Direct contact with patients	2
No contact with patients	1
Not related to patient care	0

**Table 2:** Physical risk.

Physical risk	Score
die	12
Harm	6
Treatment error	3
Discomfort	2
Delay in diagnosis and treatment	1
No problem	0

**Table 3:** Device features.

Device features	Score
Electronic equipment	2
Mechanical equipment	2
has moving parts	2
There are parts that need to be replaced regularly	2
Systematic correlation downtime	2
Must be cleaned regularly	2
There is obvious user intervention	2

**Table 4:** Safety features.

Safety performance	Score
Patient condition alarm	1
Error alarm	1
Silent light alarm	1
Fault code display	1
Continuous backup test	1
Mechanical safety protection	1
Continuous operation warning	1
Start self-test	1
Manual self-test	1

**Table 5:** Lethal status and frequency of use.

Usage frequency	Score
High	5
Higher	4
Low	2
Hardly ever	0
Direct death	5
Indirect death	3
did not cause death	0

## Data Availability

The data underlying the results presented in the study are available within the article

## References

[1] Zheng X., Liu N., Wang W. (2018). New problems and improvement ideas of medical equipment quality control. *Zhongguo yi liao qi xie za zhi = Chinese journal of medical instrumentation*.

[2] Hao Y., Heqing (2019). [Research on risk control of hysteroscopy and laparoscopy based on cloud computing].[J]. *Zhongguo yi liao qi xie za zhi = Chinese journal of medical instrumentation*.

[3] Saffarpur M., Tahmasebi Namin Y., Shaikaba Mehr A. H. (2019). Evaluation of the effectiveness of dental curing light in dental offices located in Karaj in 2016. *Alborz University Medical Journal*.

[4] Chen J., Li X., Zhong X. (2019). Third investigation and analysis of quality control situation of intensive care unit in traditional Chinese medicine hospitals in Sichuan Province. *Zhonghua wei zhong bing ji jiu yi xue*.

[5] Jose E. (2018). Villarreal‐Barajas. COMP report: CQPR technical quality control guidelines for treatment planning systems[J]. *Journal of Applied Clinical Medical Physics*.

[6] Liu Y., Yin H., Yang P. (2017). Status and tendency of clinical quality control procedure and regulation for MRI equipment[J]. *Chinese Journal of Medical Imaging Technology*.

[7] Du Y., Zhao T. (2021). Network teaching technology based on big data mining and information fusion. *Security and Communication Networks*.

[8] Zhang N., Xu B., Jia S. Y. (2018). Modeling extraction process of Salvia miltiorrhiza based on multi-source information fusion technology[J]. *Chinese Traditional and Herbal Drugs*.

[9] Shuai C., Yang Y., Zheng W. (2020). Low-altitude protection technology of anti-UAVs based on multisource detection information fusion:[J]. *International Journal of Advanced Robotic Systems*.

[10] John S. C., John A., Cuthbertson L., VanKoeveringGreen G (2017). 3D printing to repair, modify and create medical equipment in a resource limited setting. *Annals of Global Health*.

[11] Pickles S. F., Pritchard D. I. (2017). Quality control of a medicinal larval (*Lucilia sericata*) debridement device based on released gelatinase activity. *Medical and Veterinary Entomology*.

[12] Kamko Y. A. (2018). Key aspects of raising efficiency of management of equipment maintenance and repair in fuel and energy complex companies. *Safety and Reliability of Power Industry*.

[13] Zhu Y., Qiu T., Miao W. (2022). Interactive art design based on intelligent sensors and information fusion technology. *Wireless Communications and Mobile Computing*.

[14] Ouyang X., He W., Lu W. (2018). Study on 3D border surveillance system and multi-sensor information fusion technology[J]. *Bandaoti Guangdian/Semiconductor Optoelectronics*.

[15] Zhang Z., Xiong X. (2017). Research on the method of new generation of multi-sensor information fusion technology to promote the development of wisdom agriculture in ’internet +’ mode[J]. *Fresenius Environmental Bulletin*.

[16] Wang Y., Zheng G., Wang X. (2019). Development and application of a goaf-safety monitoring system using multi-sensor information fusion[J]. *Tunnelling and Underground Space Technology*.

[17] Xia J., Feng Y., Liu L. (2018). An information fusion model of innovation alliances based on the bayesian network[J]. *Tsinghua Science and Technology*.

[18] Andargoli S., Malekzadeh J. (2017). LPI optimization framework for search radar network based on information fusion[J]. *Aerospace Science and Technology*.

[19] Wu J., Su Y., Zhu Y. (2017). Real-time remaining useful life prediction of cutting tool based on information fusion[J]. *Journal of Huazhong University of Science and Technology (Nature Science Edition)*.

[20] Lu C., Wang S., Wang X. (2017). A multi-source information fusion fault diagnosis for aviation hydraulic pump based on the new evidence similarity distance. *Aerospace Science and Technology*.

[21] Pan J., Zhu H., Gao J. (2018). Study of intelligent decision making technology for relay protection setting notification based on information fusion[J]. *Dianli Xitong Baohu yu Kongzhi/Power System Protection and Control*.

[22] Wang L., Wu W., Wei G. (2018). Positioning information fusion for dual marine INSs based on grid frame[J]. *Zhongguo Guanxing Jishu Xuebao/Journal of Chinese Inertial Technology*.

